# Adipose-Derived Stem Cells for Tissue Engineering and Regenerative Medicine Applications

**DOI:** 10.1155/2016/6737345

**Published:** 2016-02-21

**Authors:** Ru Dai, Zongjie Wang, Roya Samanipour, Kyo-in Koo, Keekyoung Kim

**Affiliations:** ^1^School of Engineering, University of British Columbia, Kelowna, BC, Canada V1V 1V7; ^2^West China Hospital, West China Medical School, Sichuan University, Chengdu 610041, China; ^3^Department of Biomedical Engineering, University of Ulsan, Ulsan 680-749, Republic of Korea

## Abstract

Adipose-derived stem cells (ASCs) are a mesenchymal stem cell source with properties of self-renewal and multipotential differentiation. Compared to bone marrow-derived stem cells (BMSCs), ASCs can be derived from more sources and are harvested more easily. Three-dimensional (3D) tissue engineering scaffolds are better able to mimic the* in vivo* cellular microenvironment, which benefits the localization, attachment, proliferation, and differentiation of ASCs. Therefore, tissue-engineered ASCs are recognized as an attractive substitute for tissue and organ transplantation. In this paper, we review the characteristics of ASCs, as well as the biomaterials and tissue engineering methods used to proliferate and differentiate ASCs in a 3D environment. Clinical applications of tissue-engineered ASCs are also discussed to reveal the potential and feasibility of using tissue-engineered ASCs in regenerative medicine.

## 1. Introduction

Millions of people worldwide suffer from diseases, and the majority could be helped or cured through tissue or organ transplantation. However, deficiencies in tissues and organs are a huge challenge for medicine [1] that has resulted in the emergence of regenerative medicine, which is an interdisciplinary field involving biology, medicine, and engineering [2]. Regenerative medicine aims to repair, replace, maintain, or enhance tissue and organ functions and offers therapeutic solutions for many diseases [2, 3]. In recent years, the rapid development of biology, biomaterials, and tissue engineering has promoted the development of regenerative medicine. The traditional ways of culturing cells in a two-dimensional (2D) environment fail to allow interactions between cells and the extracellular matrix (ECM) [4]. As a result, three-dimensional (3D) biomaterial scaffolds combined with reliable sources of stem cells and biomolecules have become popular [5].

Adipose-derived stem cells (ASCs) are a mesenchymal stem cell source with self-renewal property and multipotential differentiation. ASCs can become adipocytes [6], osteoblasts [7], chondrocytes [8], myocytes [9], neurocytes [10], and other cell types [11]. ASCs also have the potential to treat various diseases, such as graft-versus-host disease [12], autoimmune-induced diseases [13, 14], multiple sclerosis [15], diabetes mellitus [16], and tracheomediastinal fistulas [17]. Compared to other types of stem cells, ASCs have two main advantages. On the one hand, ASCs can be easily accessible from subcutaneous liposuction in large numbers [18]. On the other hand, ASCs have no ethical and political issues compared to embryonic stem cells because they can be derived from autologous fat [19]. These two characteristics make ASCs become a more acceptable solution for tissue and organ transplantation in regenerative medicine and clinical studies [20, 21].

ASCs have been traditionally cultured in conventional 2D condition, which are inappropriate to mimic cell-cell and cell-environment interactions* in vivo *[22, 23]. Tissue-engineered 3D scaffolds have tremendous capacity to closely mimic* in vivo* cellular environments [24, 25]. These 3D scaffolds are generated using biofabrication methods by combining biomaterials, molecular growth factors, and extracellular matrices together to provide a 3D microenvironment for cell proliferation and differentiation, which further regulates the growth of tissues or organs [26]. In 3D scaffolds, the differentiation lineage of ASCs can be controlled by the mechanical, chemical, and other cues from microenvironment [27]. In addition to controlling differentiation, 3D scaffolds can also enhance the cell viability during proliferation [28]. Considering the benefits above, more and more attention has been paid to study ASCs within 3D scaffolds* in vitro*.

The most essential components for preparing and regulating 3D scaffolds are biomaterials and fabrication methods. Till now, many biomaterials have been utilized to grow ASCs in 3D scaffolds. ASCs localize, attach, and proliferate during* in vitro* 3D encapsulation. The ideal biofabricated scaffolds offer ASCs proper environments to facilitate their proliferation and maintain their differentiation potentials. Many key attributes of biomaterials must be considered as it closely mimics* in vivo* 3D environments: first, biomaterials should be biocompatible and do not cause a long-term immune reaction [29]; second, the biomaterials are desired to have highly porous structures with interconnected architecture to imitate the native tissue niche [30]; third, the biomaterials should have adjustable mechanical properties to regulate the cellular microenvironment. Maintaining biochemical, biomechanical, and biological properties during proliferation is also important to withstand the external environment impact [29]. With the development of biomaterials and biofabrication, many methodologies have been employed to fabricate 3D scaffolds for cell culturing, including bioprinting [31], patterning [32], self-assembling [31], and organ-on-a-chip [33]. Most of listed methodologies have been utilized to encapsulate the ASCs inside the scaffolds with the desired structure, which stimulates the differentiation of ASCs into a specific cell type for clinical application.

Current studies and clinical trails indicate that ASCs in 3D scaffolds can be a potential alternative for wound healing [34], cardiovascular grafts [35], orthopedic tissue repair [36], and plastic tissue reconstruction after surgery [37]. The success of aforementioned applications proves the great potential of ASCs to be served as a cell-based therapy for regenerative medicine. Although tissue-engineered ASCs are recognized as an attractive substitute for regenerative medicine, there are remaining problems to be solved, including the mechanisms of the interactions among ASCs, the serum-free culturing methodology, and the long-term safety. Therefore, many studies have focused on basic and animal experiments and a few clinical trials have been performed.

In this review, we discuss the characteristics of ASCs and the biomaterials and tissue engineering methods applied to regulate ASCs in 3D scaffolds. In Section 2, we discuss the characteristics of ASCs, including their background and methods to harvest and isolate ASCs. In Section 3, the biomaterials and biofabrication methods used for ASCs are discussed. In Section 4, we report current clinical cases using tissue-engineered ASCs as therapies. Finally, a brief prospective of ASCs in tissue engineering is introduced, and a short conclusion is presented.

## 2. Characteristics of ASCs

### 2.1. Background of ASCs

In general, stem cells can be divided into four categories based on their origin: embryonic [38], fetal [39], adult stem cells, and induced pluripotent stem cells (iPSCs) [40]. The characteristics of different stem cells are summarized in Table 1. Human embryonic stem cells (ESCs) are a type of stem cells derived from the inner cell mass of developing blastocysts [38] and widely used in tissue engineering and regenerative medicine because of their high capacity for differentiating. ESCs are pluripotent, can be grown into adult postnatal cells, and have a greater potential for regenerative medicine compared to adult stem cells. However, ethical problems and insufficient sources limit the applications of ESCs for clinical use [38]. Human fetal stem cells, such as amniotic fluid stem cells and umbilical stem cells, are a type of stem cells originated antenatal fetal tissue as well as postnatal fetal appendixes [39]. They are broadly multipotent and have less ethical issues compared to ESCs [41]. However, the limited sources of human fetal stem cells still restricted their applications. Induced pluripotent stem cells (iPSCs), since being discovered by Takahashi et al. in 2007 [42], have made a breakthrough in regenerative medicine. iPSCs have no ethical issues and are rich in sources. However, target cells are difficult to induce through current methodologies and technologies. Therefore, because of high availability of sources, easy accessibility, and relatively low ethical issues, the adult stem cells become an attractive and promising solution for current research and medical use of regenerative medicine.

ASCs are a mesenchymal stem cell source that can easily be isolated from adipose tissue. Similar to other stem cells, ASCs can self-renew and differentiate into other cell types in the body. They were first described by Zuk et al. in 2001 as a population of cells derived from human adipose tissue with the capability of multilineage differentiation [43]. This study opened a new window for regenerative medicine using adipose tissues. Adipose tissue belongs to the mesodermal layer in embryonic period [44] and is comprised of adipocytes and a stromal vascular fraction (SVF), which is a set of heterogeneous cells, including preadipocytes, fibroblasts, vascular smooth muscle cells, endothelial cells, macrophages, lymphocytes, and ASCs [45, 46]. Differentiation of ASCs was initially considered to be limited to mesodermal tissue only. However, recent studies have extended the use of ASCs to ectodermal and mesodermal tissues and organs [21]. More recent studies have revealed that ASCs have a variety of differentiation pathways, including adipogenesis, osteogenesis, chondrogenesis, and other lineages [11].

Bone marrow-derived stem cells (BMSCs) are the most commonly used adult stem cells. However, ASCs have many advantages over BMSCs. Firstly, the extraction procedure of BMSCs is painful, and the yield rate of cells is quite low [11], while ASCs have abundant sources that are localized in subcutaneous adipose tissue throughout the body. Besides, it is easy to obtain ASCs using the minimally invasive liposuction [47], and the percentage of obtained cells is relatively higher than other stem cell sources [47]. In addition, ASCs can be transplanted to autologous or allogeneic body safely with less implant migration and foreign body reaction [29]. Thus, ASCs become the most attractive stem cell source for tissue engineering and regenerative medicine.

### 2.2. Harvesting of ASCs

ASCs are harvested from human subcutaneous adipose tissue. Current techniques to harvest ASCs include Coleman's technique [48], liposuction [49, 50], and direct excision [48, 49]. Among them, the most popular technique is Coleman's technique. Liposuction includes conventional liposuction (tumescent) and ultrasound-assisted liposuction [49]. Iyyanki et al. showed that the yield of ASCs harvested from the abdomen through direct excision or Coleman's technique with centrifugation was higher than that from liposuction and Coleman's technique without centrifugation [48]. They also indicated that adipose tissue harvested from the abdomen contained a larger number of SVF cells than that harvested from the flank or axilla. However, Schreml et al. reported no significant difference in the number of ASCs or adipogenic differentiation potential between direct resection and liposuction [50]. In contrast, the percentage of viable cells was dramatically higher through liposuction than through direct resection [50].

Adipose tissue is widely distributed in the human body and the location influences the stem cell yield [48]. In practice, adipose tissue is usually harvested from the abdomen or the hip/thigh region [51]. Patient age is another important factor influencing ASC yield. Wu et al. showed that all age groups have similar ASCs and osteogenic paracrine activities [52]. In contrast, ASCs from infants have higher angiogenic and osteogenic capabilities than those from adults and elderly people. Another study supported this idea that proliferative activity, colony-differential potential, and population doubling are significantly different in ASCs harvested from young patients (>20 years old) and from older patients (50–70 years old) [53]. Due to the advancement of technologies, ASCs can now be acquired in large quantities using minimally invasive techniques. However, the best harvesting method to yield the largest numbers of ASCs with optimal biological function remains unclear.

### 2.3. Isolation of ASCs

Adipose tissue contains various cell types. The most widely utilized method to isolate ASCs from other cells relies on collagenase digestion, followed by centrifugation. A recent study suggested that incubating adipose tissue with 0.25% trypsin for 60 min is more cost-effective and efficient than tissue digestion with collagenase [54]. In that study, nine protocols, including collagenase, red blood cell lysis buffer solution, various trypsin concentrations, and centrifugation, were compared based on isolation rate, cell viability, expansion rate, immunophenotype, and the differentiation into adipogenic and osteogenic lineages. The results showed that trypsin-digested ASCs had similar proliferation capacity to those treated with collagenase and better osteogenic differentiation result. Thus, trypsin-based protocol is attractive for isolating ASCs considering cost and yield.

All of aforementioned methods separate primary adipocytes floating at the top from SVF accumulated at the bottom after centrifugation. SVF is a heterogeneous cell population comprised of various cells and ASCs [18]. ASCs are distinguished from other cells by morphology and immunophenotype. ASCs have fibroblast-like morphology and lack lipid droplets in the cytoplasm [29]. In addition, ASCs strongly express CD13, CD29, CD49d, CD73, CD90, CD133, MHC I, and MHC II, but they do not express CD106, which is commonly expressed on BMSCs [11].

Isolating ASCs is an important step for tissue engineering applications. Therefore, a cost-effective isolation method is essential for further applications. Comparing different protocols and determining the best one can contribute significantly to the development of adipose tissue engineering.

### 2.4. ASC's Culture and Preservation

Isolated ASCs are commonly proliferated in conventional culturing condition before 3D encapsulation. A typical culturing condition is Dulbecco's modified Eagle medium (DMEM) with 10% fetal bovine serum (FBS) and 1% antibiotics at 37°C and 5% CO_2_ in monolayer dishes [55]. However, when exposed to human body, animal-derived FBS may cause the risk of graft rejection or infection. Therefore, for clinical application, xeno-free culture media without animal-derived reagents should be developed. Lindroos et al. adopted serum-free and xeno-free media (using allogeneic human serum as a replacement) to culture ASCs and these media maintained the proliferation and differentiation of ASCs [56]. Platelet-rich plasma (PRP) has been proposed as a promising alternative for ASCs culture [57]. Atashi et al. studied the efficiency of autologous nonactivated PRP (nPRP) or thrombin-activated PRP (tPRP) (1–60%) on ASC proliferation compared to conventional 10% FBS. The results indicated that nPRP showed dose-dependent performance and its influence on ASCs was higher than FBS or tPRP without changing cell properties [57]. The results were consistent with Liao et al. [58] who proved that PRP could improve ASC proliferation while inhibiting adipogenic differentiation of ASCs in adipogenic media. Combining with biofabricated 3D scaffolds, serum-free and xeno-free media have great potential to provide the less risky and cytoactive condition for the off-the-shelf therapies.

Preservation of ASCs is also essential for tissue engineering and regenerative medicine. ASCs can be preserved in the conventional cryopreservation media including 90% FBS and 10% dimethyl sulfoxide (DMSO) [59]. For the off-the-shelf therapies in clinical practice, preservation methods are required to store large quantity of ASCs and maintain their properties in the long term. Recently, De Rosa et al. employed low DMSO cryopreservation methods to reduce toxicity of DMSO in room temperature. In this study, threalose was used as a replacement of DMSO. A solution combing 4% DMSO, 6% threalose, and 90% FBS resulted in maintaining the stemness and differentiation property of ASCs [59]. Miyamoto et al. also found that ASCs kept in CELLBANKER 2 and DMEM/Ham's F-12 medium with 10% DMSO, 0.1 mol/L maltose, and 1% sericin performed better in terms of proliferation and differentiation capability comparing with standard protocols [60].

## 3. ASC's Growth and Differentiation on Biofabricated 3D Scaffolds

Biomaterials have been proved to affect the proliferation and differentiation of stem cells (SCs) by controlling chemical compositions and physical properties (e.g., mechanical properties and microstructural patterns) [66]. On the one hand, the chemical composition significantly affects the differentiation potential. For instance, collagen, which is abundant* in vivo* environments, can interact with SCs via integrin binding, while alginate, which is a seaweed-derived anionic polysaccharide and does not exist in native ECMs, cannot directly interact with stem cells [67]. Therefore, collagen can support more differentiation lineages of SCs compared to alginate. SCs can be differentiated to many different lineages (e.g., skin, bone, cartilage, tendon, ligament, lung, and nerve), when encapsulated in collagen [68]. On the other hand, physical properties also play an essential role in regulating SC's differentiation. For example, when encapsulated in polycaprolactone (PCL), SCs tended to differentiate to mesenchymal lineages (e.g., bone, tendon, and cartilage) since the stiffness of PCL mimicked* in vivo* mechanical property of mesenchymal tissues [67]. In addition, it has shown that the precise control of scaffold microstructural patterns using nanobiotechnology can affect the differentiation of SCs [69]. SCs could differentiate to osteoblast rather than adipocyte when the size of pattern seeding SCs was increased. Taken together, chemical and physical effects of the SC microenvironments created by biomaterials can significantly affect SC differentiation.

### 3.1. Tissue-Engineered 3D Scaffolds for ASC Culture

Fabricating proper scaffolds that facilitate ASC proliferation is important for the tissue engineering applications. The characteristics of scaffolds can be adjusted by encapsulating nanostructures, using different types of biomaterials, or modifying the mechanical or electrical properties of biomaterials through coating.

Silica nanoparticles (NPs) have been used to increase proliferation of ASCs by activating extracellular signal-related kinase (ERK) 1/2 [70]. The ERK 1/2 signaling pathway is a mitogen-activated protein kinase (MAPK) pathway related to biopolymer-induced proliferation of stem cells. ASCs were seeded into a 96-well plate with DMEM and 1% FBS containing silica NPs and silica microparticles (MPs). It was reported that the ASC proliferation rate increased significantly in the presence of the silica NPs, while no obvious change in proliferation was observed with silica MPs, indicating that silica composites promoted proliferation of ASCs and that the MAPK pathway is a possible mechanism regulating proliferation. Another study showed that the proliferation rate of ASCs cultured in media containing 2 or 4 *μ*M silicon was significantly greater than that in control medium. The improved mechanical strength of the medium with silicon may contribute to this result [71]. Therefore, scaffolds containing NPs for tissue engineering may enhance ASC growth and single component silica-derived NPs could be useful for the scaffolds in stem cell therapy [70].

Natural polymers also benefit the proliferation of ASCs. Hyaluronic acid (HA) is an effective natural biomaterial to increase ASC proliferation. HA scaffolds formed porous structure, which was commonly regarded as a preferable culturing model for cell proliferation. Besides, ASCs tend to form small dispersed aggregates in HA scaffolds, and the small-sized cell aggregates facilitated metabolic exchange. The above factors may account for the enhanced proliferation of ASCs [72]. Other nature-derived scaffolds, such as collagen-HA scaffold [73], and type I collagen scaffold [74], have also been reported to facilitate the proliferation of ASCs.

**Figure 1:**
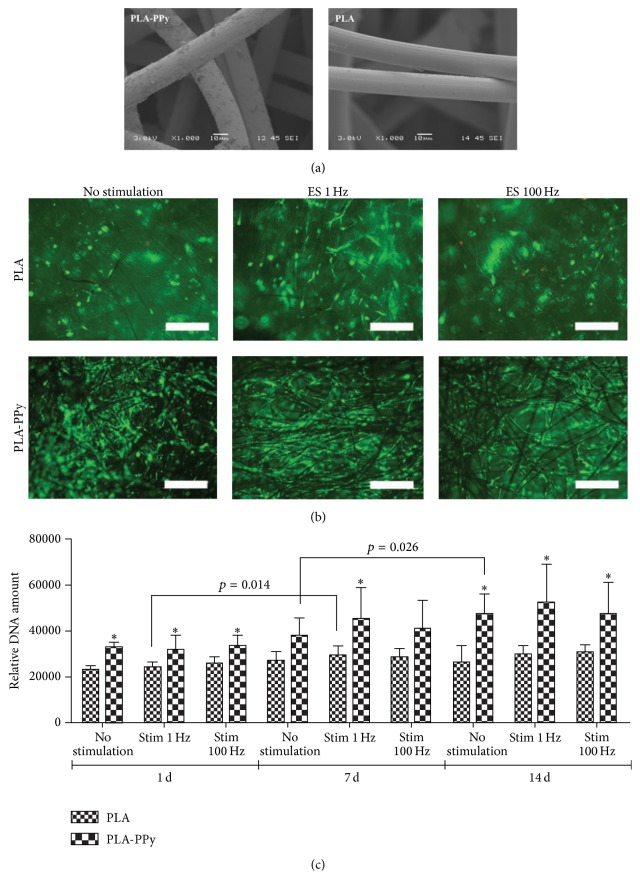
PPy-coated PLA scaffolds are able to enhance ASC proliferation through electrical stimulation. (a) Scanning electron microscopy image of PLA-PPy scaffold (left) without sputtered gold layer and PLA scaffold (right) with 20 nm gold coating. (PLA scaffold could not be imaged without coating due to the heavy electrostatic.) (b) Representative images of the viable (green) and dead (red) ASCs seeded in PLA and PLA-PPy scaffolds at day 14 (scale bar: 500 *μ*m). (c) Relative DNA content of ASCs cultured for 1, 7, and 14 days in PLA and PLA-PPy scaffolds (in the form of mean ± standard deviation, *n* = 3, ^*∗*^ *p* < 0.05) (adopted from [61]).

Highly conductive scaffold could also upregulate the proliferation of ASCs in synthetic polymers. The proliferation of ASCs in polypyrrole-coated polylactide (PLA-PPy) and PLA scaffolds has been investigated [61]. PPy was an electrically conductive material, while PLA was electrically insulated. In this study, PLA was coated with PPy to regulate its conductivity properties under different electrical stimulation.  Figure 1(a) presents the scanning electron microscope images of the microstructures of PLA and PPy-coated PLA. Electrical stimulation may serve as a potential factor to stimulate ASCs proliferation as well as differentiation like most molecular factors do.  Figure 1(b) shows the representative fluorescent images of the ASCs cultured in both scaffolds after 14 days. It is clear that the number of ASCs and the attachment rate of ASCs in PLA-PPy scaffold are significantly higher than PLA scaffold, revealing that the proliferation rate of ASCs was higher in PLA-PPy scaffolds (no stimulation, 1 Hz stimulation, or 100 Hz stimulation) than that in PLA scaffolds. The DNA content analysis results are also consistent with the fluorescent images. The amount of DNA increased significantly when using PLA-PPy scaffolds (Figure 1(c)). Thus, it can be concluded that conductive scaffold benefits the proliferation of ASCs. However, judging from Figures 1(b) and 1(c), the proliferation rate under different electrical stimulation did not differ significantly. This may be due to the fact that PPy is an electronic conductor both in air and in medium and can regulate suitable stimulation current to cells regardless of external electrically stimulation [61]. However, further study is required to quantitatively analyze the effects of electrical stimulation.

Many other biomaterials have been employed for culturing ASCs in 3D environments, such as chitosan [75], silk [76], alginate [77, 78], and natural and synthetic calcium phosphate [7]. Various scaffolds with different tissue properties including geometry, porosity, stiffness, surface characteristics, and composition have been tested. External stimulus is another factor to influence ASC proliferation. Jeong et al. found that low-dose ultraviolet B (UVB) radiation did not affect ASC proliferation, while high-dose UVB reduced the proliferation of ASCs [79]. In addition, many researches showed that magnetism could enhance ASC differentiation, while its ability to enhance ASC proliferation was not confirmed [80, 81].

### 3.2. Differentiation of ASCs in 3D Scaffolds

ASCs have great differentiation potential. In this section, different tissue engineering methods are presented for inducing various types of differentiation.

#### 3.2.1. Adipogenesis

Adipogenesis is the original ASC differentiation pathway. ASCs are capable of differentiating into adipocytes on particular scaffolds when combined with appropriate cues. Both alginate and alginate/gelatin microspheres seeded with ASCs facilitate adipogenesis, whereas an alginate/gelatin matrix supports the adipogenesis pathway better than that of alginate alone [78]. PDM combined XLHA scaffolds also benefit adipogenesis, and mature adipocytes occur more frequently in the central region of these scaffolds [82]. ASCs seeded in micromolded resections of agarose hydrogel form tissue spheroids after 3 weeks of culture, and the cells were multipotent for the adipogenic lineage [83]. ASCs cocultured with human umbilical vein endothelial cells (HUVEC) in collagen/alginate microspheres could be used as a biomimetic physiological model. Abundant lipid accumulation and morphological changes representing the adipogenic differentiation were detected after 17 days of coculturing [84].

**Figure 2:**
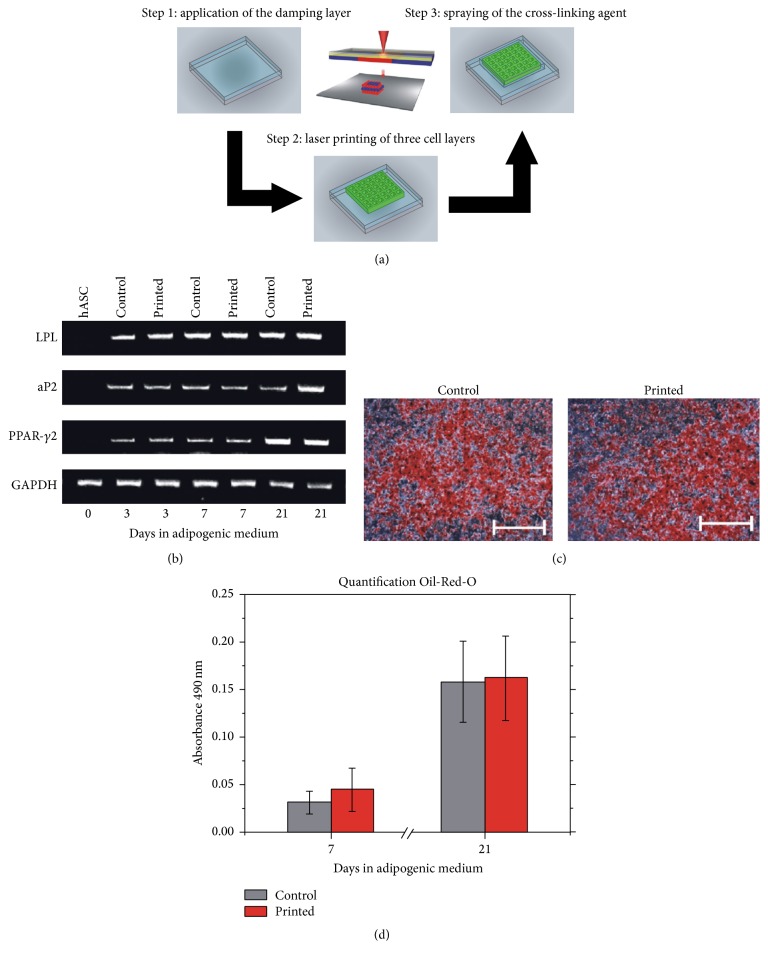
Laser-assisted bioprinting helps in the proliferation and differentiation of ASCs. (a) Three-dimensional scaffolds were fabricated via laser-assisted bioprinting. During printing, the laser pulse was applied to melt a certain area in the energy absorbing layer (red layer). The suspended biomaterial prepolymers below that area fell due to the loss of support from the absorbing layer. The small units formed droplets that were collected by the bottom substrate. The prepolymers were cross-linked using a cross-linking agent. (b) Lipase (LPL), adipocyte fatty acid-binding protein (aP2), and peroxisome proliferator-activated receptor (PPAR-*γ*2) expression were compared on printed and nonprinted control ASCs compared to unstimulated ASCs after 3, 7, and 21 days of incubation. (c) Oil Red-O staining indicated similar lipid accumulation in the cytoplasm of the printed and nonprinted groups (scale bar: 500 *μ*m). (d) Equal lipid quantities were detected in both groups (*α* = 0.05) (adopted from [62]).

Bioprinting methods also help to maintain the differentiation potential of ASCs. ASCs have been encapsulated inside a 3D scaffold using laser-assisted bioprinting [62] (Figure 2(a)). In this study, alginate was employed as the scaffolding materials. Alginate is a natural hydrogel and has been widely used for tissue engineering applications. It has been also reported that ASCs could be differentiated to other cell types in alginate scaffolds [77, 78, 85]. During printing, a laser pulse was applied to the energy absorbing layer (red layer), generating a high-pressure bubble and propelled the suspended cells below that area. The small falling unit formed a droplet that was collected in the bottom substrate, and the prepolymers were cross-linked with a cross-linking agent. ASCs encapsulated scaffolds and ASCs cells in 2D were cultured in 24-well plates for 21 days in DMEM containing 10% FBS, 1 *μ*M dexamethasone, 10 *μ*g mL insulin, 0.5 mM 3-isobutyl-1-methylxanthine, and 0.2 mM indomethacin. The expression of adipogenic markers, including lipase (LPL), adipocyte fatty acid-binding protein (aP2), and peroxisome proliferator-activated receptor (PPAR-*γ*2) as well as lipid accumulation in the cytoplasm, was evaluated at days 3, 7, and 21 to investigate the degree of cell differentiation. Gene expression was measured by the real-time reverse transcriptase-polymerase chain reaction (RT-PCR). As shown in Figure 2(b), LPL, aP2, and PPAR-*γ*2 expression levels in the printed and nonprinted groups were similar after 3, 7, and 21 days. However, the expression levels of LPL, aP2, and PPAR-*γ*2 were promoted compared to those in unstimulated ASCs, indicating that bioprinted 3D scaffold may upregulate the differentiation of ASCs. The Oil Red-O staining result indicated similar lipid accumulation in the cytoplasm of the bioprinted and nonprinted groups (Figure 2(c)). The quantitative assessment of lipid accumulation using radiation absorbance also revealed that lipid quantities were equal in the two groups after 7 and 21 days. This study verified that laser-assisted bioprinting has no significant negative effect on ASC proliferation and differentiation. Therefore, laser-assisted bioprinting is a promising manufacturing method for ASCs encapsulated 3D scaffolds for tissue engineering applications.

#### 3.2.2. Osteogenesis

**Figure 3:**
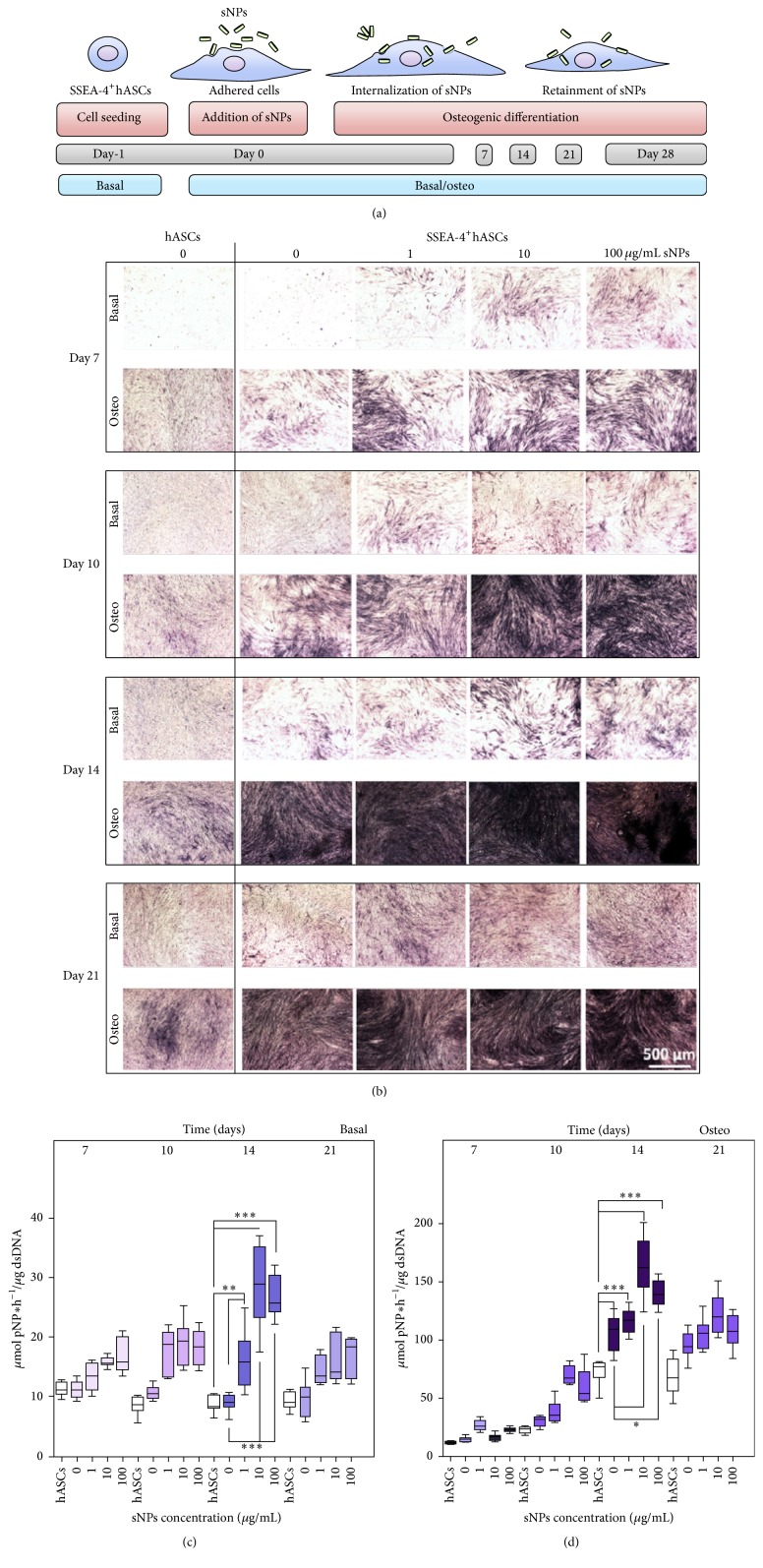
Culturing ASCs with bioactive silicate nanoplatelets promotes the osteogenic differentiation of ASCs. (a) Experimental procedures used to induce osteogenic differentiation. SSEA-4^+^ adipose-derived stem cells (ASCs). SSEA-4^+^ ASCs were seeded in basal medium for 1 day, and silicate nanoplatelets (sNPs) were added to the basal medium. The samples were incubated either in basal medium or under osteogenic differentiating conditions. Samples were collected on days 7, 14, 21, and 28 to analyze differentiation. (b) Qualitative analysis of alkaline phosphatase (ALP) in SSEA-4^+^ ASCs on days 7, 10, 14, and 21 cultured in basal and osteo media, compared to ASCs. The dark purple color increased with sNP concentration level and osteo medium presented a darker purple color. (c) Quantitative analysis of ALP in SSEA-4^+^ ASCs in basal medium with sNPs. The presence of sNPs enhanced ALP activity on day 14, compared to that in ASCs. (d) Quantitative analysis of ALP in SSEA-4^+^ASCs in osteo medium with sNPs. The presence of sNPs enhanced ALP activity on day 14, compared to that in ASCs (adopted from [63]). ^*∗*^ *p* < 0.05, ^*∗∗*^ *p* < 0.01, and ^*∗∗∗*^ *p* < 0.001.

ASCs can differentiate into osteocytes as well. It has been found that PLA-PPy scaffolds can provide higher alkaline phosphatase (ALP) activity levels, which benefit the early osteogenic differentiation of ASCs [61]. In another study, a silicon dioxide gel was fabricated to serve as the medium for ASC culturing. Expression of the osteogenic genes osteocalcin and osteopontin reached the highest in medium containing 2 *μ*M silicon ions. The Alizarin Red-S staining of ACSs indicated that, with 2 *μ*M silicon ions in the medium, there are maximum number of differentiated cells [71]. Mihaila et al. reported that combining ASCs with bioactive silicate nanoplatelets (sNPs) promotes osteogenic differentiation [63]. In their study, SSEA-4^+^ ASCs, which is a subset of ASCs with higher differentiation potentials, was utilized. As shown in Figure 3(a), SSEA-4^+^ ASCs were harvested from human subcutaneous abdominal tissue and isolated and selected from SVF. SSEA-4^+^ ASCs were cultured in basal medium for 1 day and then sNPs were added and incubated in either basal or osteogenic media. Samples were collected on days 7, 14, 21, and 28 to analyze expressed ALP, which appeared in early osteogenic differentiation and considered as the marker of osteogenic differentiation. The qualitative level of ALP was assessed by staining the fixed samples with nitro-blue tetrazolium/indolyl phosphate. The quantitative level of ALP was determined by an adapted end-point colorimetric procedure based on the p-nitrophenol assay. As shown in Figure 3(b), the dark purple color became more intense with the increasing of NPs concentrations and peaked on day 14. The osteo medium presented a darker purple color than that of the basal medium, which was consistent with the quantitative results shown in Figures 3(c) and 3(d). Taken together, these results indicate that the sNPs significantly promoted ALP activity in SSEA-4^+^ ASCs (*p* < 0.05), compared to the ASCs cultured in the same condition and upregulated osteogenic differentiation in both basal and osteogenic media.

Many other studies also revealed that the method regulates osteogenesis of ASCs. External stimulation as in the form of magnetic actuation was reported to influence osteogenic differentiation of ASCs [80]. In the study, Lima et al. combined magnetic nanoparticles with ASCs and cultured cells in osteogenic medium up to 28 days with or without external magnetic stimulation. The results in days 14 and 21 revealed that the alizarin red staining against calcium deposits was more intense and widespread. In addition, polyglycolic acid (PGA) mesh scaffolds could induce osteogenesis but pretreatment with osteo-induction factors did not further increase osteogenesis [86]. The mechanical properties of hydrogels could also affect osteogenesis process. ASCs had been cultured and differentiated in poly- (ethylene glycol-) diacrylate (PEGDA) of different molecular weights and concentrations. Mineralization and osteocalcin gene expression were examined as indicators of osteogenesis. The results showed that osteogenesis of ASCs increased with matrix stiffness, indicating that a stiff matrix mimicking the native microenvironment of bone is beneficial for osteogenesis [87].

#### 3.2.3. Chondrogenesis

Chondrogenesis is another common pathway of multipotent mesenchymal cells. HA scaffolds also have the capacity to induce chondrogenesis in ASCs [72]. In this study, researchers prepared poly ethylene glycol diglycidyl ether- (PEGDG-) cross-linked porous 3D HA scaffolds and investigated their feasibility for differentiating ASCs into chondrocytes using cell sulfated glycosaminoglycan content. The results showed that chondrogenic differentiation of ASCs in the scaffolds was higher than that in micromass culture. In another study, ASCs were cultured in alginate microspheres, and upregulation of cartilage specific genes, including transforming growth factor-*β*, collagen type-X, and cartilage oligomeric matrix protein, was observed [77]. In another study, plasmid DNA (pDNA) containing SOX trio genes was incorporated into PLGA scaffolds with ASCs [88]. Increases in COL2A1 gene expression and protein were seen in SOX trio pDNA-incorporated scaffolds compared to that in the control group, indicating the upregulated effects of pDNA to chondrogenesis. Electromagnetic field also promotes the chondrogenic differentiation of ASCs [81]. Chen et al. found that electromagnetic field could improve chondrogenic differentiation while not affecting cell viability.

#### 3.2.4. Other Lineages

**Figure 4:**
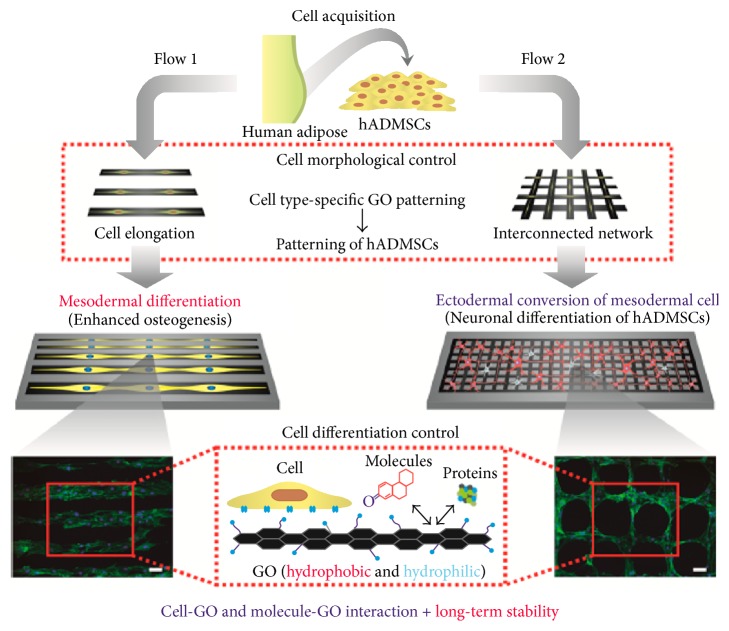
Graphene pattern controlled ASCs differentiation. The schematic illustrates the steps taken to modulate the morphology of ASCs using graphene oxide patterns, which further guide the differentiation of ASCs. Fluorescent images are F-actin stained ASCs. (Scale bar = 100 *μ*m.) Flow 1: the line pattern enhances the osteogenesis of ASCs. Flow 2: the mesh pattern enhances the neurogenesis of ASCs (adopted from [64]).

ASCs can follow other differentiation pathways in biofabricated scaffolds mimicking specific* in vivo* environment. For example, ASCs can become smooth muscle cells and endothelial cells [35]. In this study, a customized electrospun scaffolds using electrospun nano to microscale collagenous and elastic fibers were created to mimic the natural cardiovascular environment and induce ASC differentiation. After two weeks' culturing, ASCs migrated into the scaffolds, interconnected with the surrounding environment, and developed into endothelial cells and smooth muscle cells under different culture environments. Desiderio et al. showed that ASCs can also form a human skeletal muscle tissue* in vitro* through the culture with cross-linked hyaluronic acid scaffold [89]. Gao et al. revealed that using ASCs could obtain neuron-like cells through seeding ASCs in photocurable 3D chitosan and gelatin scaffolds [90]. ASCs are capable of becoming cardiomyocytes for cardiovascular tissue engineering [91]. In detail, two engineered scaffolds, aligned polycaprolactone (PCL) nanofibrous electrospun scaffolds and random PCL nanofibrous scaffolds, were used to proliferate and differentiate ASCs to cardiomyocytes. The results revealed that the aligned PCL nanofibrous scaffolds were more appropriate to induce differentiation of cardiomyocytes since it guided the growth direction of ASCs. A recent study from Kim et al. further revealed the potential to use nanostructure for manipulating and guiding ASC differentiation [64]. In this study, graphene oxide (GO) was precisely patterned to control cell morphology, which significantly affected ASC differentiation. The result shows that the line pattern of GO, which closely mimics the environment of osteoblasts, enhanced the osteogenesis of ASCs (Figure 4, Flow 1). In addition, the mesh pattern of GO upregulated neuronal differentiation of ASCs (Figure 4, Flow 2). This study demonstrated that a combinatorial method combining nanomaterials and biofabrication could accurately and effectively control the differentiation of ASCs.

We summarized representative biofabrication and tissue engineering methods employed to ASCs in Table 2. In summary, the differentiation capacity and the fate of ASCs is closely related to the characteristics of the microenvironment, including the mechanical properties of scaffolds, the existence of additional inducing factors, and the alignment of the microstructures.

### 3.3. Possible Mechanism to Regulate ASC's Proliferation and Differentiation

The proliferation and multiple lineage differentiation capabilities of ASCs are elaborated in many previous articles. Despite their promising application in tissue engineering and regenerative medicine, the mechanisms of ASC's behaviors remain unclear. Some possible mechanisms have been proposed to address the proliferation and differentiation properties of ASCs.

#### 3.3.1. Growth Factors

Growth factors have been acknowledged to affect cell transition [110] and stem cell differentiation [111]. Several growth factors were reported to involve ASC proliferation and differentiation. Fibroblast growth factor-2 (FGF-2) can promote the proliferation of ASCs [92]. FGF-2 can also induce endotheliogenesis [92], osteogenesis [93], and chondrogenesis [94] of ASCs. Vascular endothelial growth factor (VEGF) has been used to promote capillary formation and induce osteogenic differentiation [95]. Platelet-derived growth factor- (PDGF-) BB was able to prompt ASCs proliferation [96], angiogenesis [97], and their migration toward the tumor-conditioned medium [98]. Bone morphogenetic protein (BMP-2) was used to enhance osteogenesis [99] and chondrogenesis [100]. It is also reported that transforming growth factor- (TGF-) *β*1 can promote ASC proliferation [101]. The effect of the growth factors on ASCs is summarized in Table 3.

#### 3.3.2. MicroRNAs

Recently, several studies have focused on investigating the role of microRNAs (miRNAs) in regulating proliferation and differentiation capabilities in ASCs. miRNAs are a species of small noncoding RNAs of 19–23 nucleotides in length [112]; miRNAs are known to negatively regulate gene expression by translational repression or inhibiting protein synthesis of target miRNAs [113]. Several recent studies indicated that miRNAs are involved in maintaining self-renewal, proliferation, and multipotential differentiation of ASCs (Table 4). For example, the involvement of miR-26a in osteogenic differentiation of ASCs was demonstrated by Luzi et al. [102]. The expression level of miR-26a was relatively high in late stages during osteogenic differentiation, suggesting that miR-26a was a positive regulator for the osteogenesis of ASCs. Kim et al. also reported that miR-196a was upregulated during osteogenic differentiation of ASCs [103]. The alteration of miR-138 and miR-21 expression during adipogenesis of ASCs suggested that those two miRNAs played an important role in the adipoblast differentiation [105, 106]. Another study showed that the level of miR-194 targeting Sox5 was decreased during chondrogenic differentiation of ASCs, whereas the upregulation of Sox5 inhibited chondrogenesis [108]. In addition, Chen et al. reported that miR-27b was related to tolerogenic response of ASCs [109]. In this study, they discussed the rat tolerogenic orthotopic liver transplantation (OLT) and rejection OLT models. It was found that miR-27b expression in the ASCs from tolerant recipients was elevated, compared to those of rejecting recipients. Taken together, the investigation of miRNAs' role in regulating ASC's behaviors provides us with information to better understand the self-renewal, proliferation, and differentiation capabilities of ASCs.

#### 3.3.3. Extracellular Signal-Related Kinase (ERK) Signaling Pathway

Activation of extracellular signal-related kinase (ERK) signaling pathway is a well-known mechanism to regulate ASC proliferation and differentiation. ERK belongs to the mitogen-activated protein kinase (MAPK) family which is a conserved family of serine/threonine protein kinases. External stimuli, such as hormones, molecular factors, physical stimuli, and environmental changes, act on target receptors of ASCs and this cell response activates ERK. The activated phosphorylated ERK then initiates a cascade of downstream events related to ASC's biological behaviors, especially differentiation property. The role of ERK signaling pathway in enhancing ASC proliferation capacity has been well discussed in the previous section. Besides, ERK signaling pathway is also known to involve ASC osteogenic differentiation. Liu et al. reported that the blockage phosphorylation level of ERK induced by a specific ERK inhibitor, PD98059, reduced the osteogenic differentiation of ASCs in a dose-dependent manner [114]. In addition, they also revealed that the combination of dexamethasone led to adipogenic differentiation conversely [114]. Kim et al. reported that MAPK pathway also involved TGF-*β*1 signaling which was believed to induce chondrogenic differentiation of ASCs. In addition, TGF-*β*1 signaling activated SMAD that was related to increasing chondrogenic activity of ASCs [115]. While ERK signaling pathway may be advantageous to induce the osteogenesis of ASCs, the role of ERK signaling pathway in other lineage differentiation and self-renewal capacity of ASCs remains unclear. Moreover, it has been reported that MAPK signaling pathway could control cancer development and tumorigenesis [116]. A group of researchers have confirmed the effect of MAPKs in liver cancer [117], brain tumors [118], prostate cancer [119], glioma [120], head and neck squamous carcinoma cells [121], thyroid tumor [122], and breast carcinoma [123]. Therefore, it is controversial to regulate ERK signaling pathway during ASC proliferation and differentiation.

## 4. Clinical Applications of Tissue-Engineered ASCs 

ASCs are being vigorously studies in the laboratory now, but a few clinical trials of ASCs have been reported compared to those for BMSCs. The official clinical trial website (https://clinicaltrials.gov/, keyword: adipose derived stem cells) revealed 125 stem cell studies, excluding unknown status. Among them, 35 trials have been completed (Table 5). Two are ongoing Phase IV clinical trials. One trial is designed to determine whether ASCs are effective for females with premature ovarian failure. The other trial was designed to evaluate transplantation of autologous fat with adipose-derived regenerative cells in patients with functional and cosmetic breast deformities after segmental mastectomy or quadrantectomy (lumpectomy). However, no result or relevant study was found at the website. Although some clinical cases have used tissue-engineered ASCs as a potential therapeutic solution, clinical trials investigation of the long-term effects of ASCs are still in progress.

**Figure 5:**
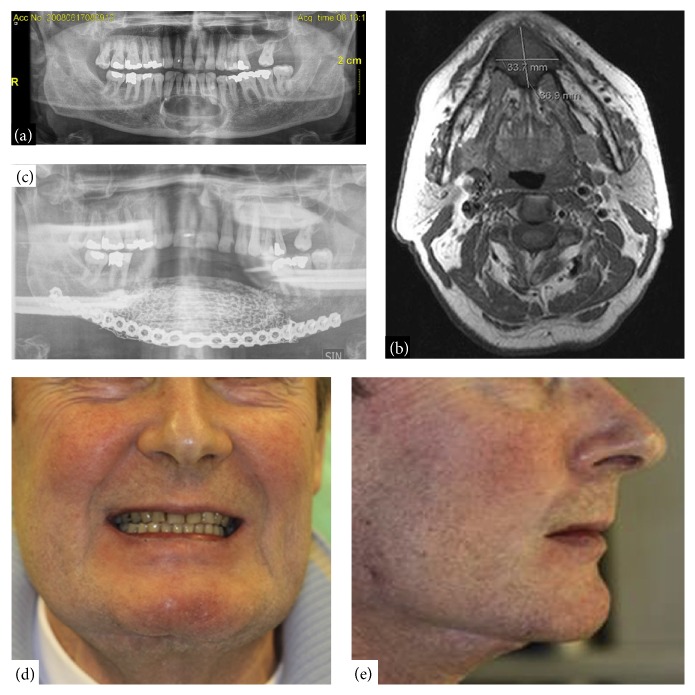
Tissue-engineered ASCs could be utilized to treat a large anterior mandibular defect. (a) X-ray image shows a nearly 10 cm recurrence of an ameloblastoma in the parasymphyseal area of the anterior mandible. (b) T1-weighted magnetic resonance image shows a large tumor (ameloblastoma) in the anterior mandible. (c) X-ray image shows the six implant fixtures inserted into the grafted site. (d) The reconstructed chin taken from the front. (e) The reconstructed chin taken from the lateral side (adopted from [65]).

Great successes in the laboratory level application have suggested the promising effects of ASCs-based in a variety of clinical condition. A recent clinical case reported that tissue-engineered ASCs could be utilized to treat a large anterior mandibular defect [65]. In this case, a 55-year-old man had a third 10 cm ameloblastoma recurrence in the parasymphyseal area of the mandible (Figures 5(a) and 5(b)). The defect site was treated with a tissue-engineered construct that combined *β*-tricalcium phosphate (*β*-TCP) granules, recombinant human bone morphogenetic protein-2 (BMP-2), and autologous ASCs. Adipose tissue was harvested from the anterior abdominal wall of the patient, and the ASCs were isolated by collagenase and expanded for 21 days in DMEM with 15% autologous serum without antibiotics* in vitro*. The ASCs was confirmed through cell surface marker expression and analyzed for osteogenic differentiation potential. Before seeding the cells, scaffolds containing *β*-TCP granules (porosity, 60%; granule size, 1.4–2.8 mm) were fabricated for cell attachment. The *β*-TCP granules were incubated for 48 hours in basal medium containing 12 mg rh-BMP-2. Then, the combined cell-biomaterial scaffolds were incubated and transported to the operating theater. A medical skull model manufactured using computed tomography data was fabricated before transplantation to fit the patient-specific reconstruction plate and titanium mesh. The mandibular bone was resected, and the defect site was replaced with the reconstruction plate and titanium mesh. Then, the cell encapsulated biomaterial scaffolds were added to fill the gaps, and six dental implant fixtures were used to attach the mesh. Five of six implanted fixtures allowed osseointegration (Figure 5(c)). The patient was satisfied with the appearance and function of the implanted borne during the 3-year follow-up (Figures 5(d) and 5(e)). This case shows that tissue-engineered ASCs offer a promising replacement for large bone defects [65]. Another clinical case enrolled four patients (three women and one man) with cranial defects on the right side, and all underwent ASC-based cranioplasty [124]. ASCs were also seeded in *β*-TCP phosphate granules. The follow-up indicated no clinically relevant postoperative complications, and the patients were satisfied with their outcomes. A cell-assisted lipotransfer technique has been employed in other clinical cases to treat facial lipoatrophy [125], cosmetic breast augmentation [126], and breast implant complications [127].

Despite ASC's positive effects in tissue engineering and regenerative medicine, many researchers reported that ASCs exhibited carcinogenic potentials. ASC enriched fat tissue grafts can be used as autologous and allogeneic transplantation for soft tissue reconstruction followed by mastectomy to reduce cosmetic and psychological problems [128]. However,* in vitro* and animal studies reported that ASCs can interact with tumor cells and induce tumor progression [129]. Yu et al. transplanted ASCs together with tumor cells subcutaneously or intracranially into BALB/c nude mice to observe tumor outgrowth [130]. The result indicated that coculture of ASCs with H460 or U87MG cells promoted tumor cell proliferation. Coinjection of ASCs with H460 or U87MG cells increased tumor cell viability* in vivo* and induced the apoptosis of normal cells. Conditioned medium from ASCs inhibited hydrogen peroxide-induced cell death of H460 or U87MG cells. These findings suggested that ASCs with H460 or U87MG cells promoted tumor growth in the nude mice. Chandler et al. also conducted a study to investigate the possible mechanism of the interaction between ASCs and tumor cells [129]. They found that tumor-secreted soluble factors from a breast tumor inhibited adipogenic differentiation but increased the proliferation, proangiogenic factor secretion, and myofibroblastic differentiation of ASCs. This changed behavior of ASCs is similar to the characteristics of breast tumors. The result was further confirmed by orthotropic mouse studies. On the contrary, Cousin et al. reported that ASCs could inhibit tumor viability and proliferation [131]. A coculture system with ASC-conditioned medium and pancreatic tumor cells was able to enhance pancreatic tumor death both* in vivo* and* in vitro*. This inhibitory effect mediated by ASCs was also found in liver, prostate, and colon cancers. Taken together, the carcinogenic potential using ASCs in transplantation remained unclear and reported results are controversial. Therefore, clinical trials using ASCs should be carefully considered by the medical history of patients, especially for the patients who has been diagnosed with any cancer previously.

## 5. Challenges and Future Perspectives

ASCs are good candidates for tissue repair and regeneration for plastic and reconstructive surgery because of their rich source and easy access. However, adipose tissue engineering is far from an “off-the-shelf” product. Before ASCs can be translated to clinical practice, a number of problems must be solved. First, industrialized xeno-free culture media without animal-derived reagents have not been well established. Although some labs have adopted serum-free and xeno-free media to culture ASCs. These media are not “off-the-shelf” products. To fully recognize the safety and efficiency of these media, further studies* in vivo* must be performed. Second, a well-defined preservation method maintaining ASCs properties in the long term is also of impact for ASCs' application in tissue engineering and regenerative medicine. Third, a few studies have considered the mechanisms of the interactions among ASCs, biomolecular growth factors, and biofabricated scaffolds. Understanding these mechanisms involved in ASCs proliferation and differentiation is much important for further clinical applications. Finally, as few clinical trials have been performed to investigate tissue-engineered ASCs, their long-term safety remains uncertain. At present, long-term safety issue is the biggest challenges of ASCs-based regenerative medicine. It is necessary to conduct more systematic studies to confirm that ASCs can be utilized as a standard therapeutic tool in regenerative medicine. In addition to safety issue of ASCs, the biomaterials also needed more long-term* in vivo* experiments. Although biomaterials are biocompatible, most of them are also derived from animal sources (e.g., collagen from rat tail and gelatin from porcine skin) and have the possibility to induce an immune reaction in the long term. Moreover, with the degradation of biomaterial in the body over time, the fraction may serve as host antibodies causing severe immune reactions. In summary, although there are a number of challenges existing, ASCs are still a very promising method in regenerative medicine with a bright future because of their rich source, relatively simple process of accessing and isolating, and their multipotentials of differentiation. In the near future, adipose tissue engineering may become “off-the-shelf” products for various diseases and benefit millions of people.

## Figures and Tables

**Table 1:** Characteristics of different stem cells.

Category	Origin	Advantages	Disadvantages	References
Human embryonic stems cells (hESCs)	Human blastocysts	Pluripotent; nonimmunogenic	Insufficient sources; ethical and religious debates	[38]

Human fetal stem cells (hFSCs)	Fetal tissue (i.e., fetal blood from umbilical cord and amniotic fluid) Fetal appendixes (i.e., fetal membranes and placenta)	Broadly multipotent; nonimmunogenic Less ethical and religious debates	Insufficient sources	[39, 41]

Adult stem cells	Adult tissues, such as bone marrow, adipose tissue, and skin	Multipotent; little ethical and religious debates; abundant sources; easily accessible	Relatively difficult to expand *in vitro*; limited use in clinical practice	[18, 19]

Induced pluripotent stem cells (iPSCs)	Somatic cells	Pluripotent like ESCs; no ethical and religious debates; abundant sources	Difficult to induce; abundant sources	[40, 42]

**Table 2:** Representative biofabrication and tissue engineering methods for ASCs culturing and differentiation.

Methods	Materials	Applications	References
Porous scaffolds	Hyaluronic acid (HA)	Proliferation and chondrogenesis	[72]
Microfluidics	Alginate/gelatin	3D cell encapsulation and adipogenesis	[78]
Porous scaffolds	Chitosan	Neurogenesis	[90]
Porous scaffolds	Polycaprolactone (PCL) nanofiber	Cardiomyogenesis	[91]
Bioprinting	Alginate	Adipogenesis	[62]
Nanostructure	Laponite silicate nanoplatelets	Osteogenesis	[63]
Porous scaffolds	Chitosan film	Hepatogenesis	[75]
Porous scaffolds	Poly-lactide-co-glycolide	Chondrogenesis	[88]
Porous scaffolds	Silk fibroin	Adipogenesis	[76]
Nanostructure	Graphene oxide	Osteogenesis Neurogenesis	[64]

**Table 3:** The effect of growth factors to ASCs.

Types of growth factors	Function	Reference
FGF-2	Promoting ASCs proliferation, endotheliogenesis, osteogenesis, and chondrogenesis	[92–94]
VEGF	Promoting angiogenesis and osteogenesis	[95]
PDGF-BB	Promoting ASCs proliferation, angiogenesis, and migration toward the tumor-conditioned medium	[96–98]
BMP-2	Promoting osteogenesis and chondrogenesis	[99, 100]
TGF-*β*1	Promoting ASCs proliferation	[101]

**Table 4:** miRNA expression of ASCs in culturing and differentiation.

Differentiation of ASCs	Involved miRNAs	Reference
Osteogenesis	miR-26a, miR-196a, and miR-138	[102–104]
Adipogenesis	miR-21, miR-138, and miR-27	[105–107]
Chondrogenesis	miR194	[108]
Proliferation	miR-196a	[103]
Tolerogenic response	miR-27b	[109]

**Table 5:** List of clinical trials using ASCs.

Condition	Number	Phase	Study design	Study type	Reference
Breast reconstruction	2	Phase 2	ASCs enriched fat graft	Interventional	NCT01771913
		Phase 4	Autologous SVFs	Interventional	NCT00616135

Lipoatrophy	1	Phase 1	SVF-enriched fat graft	Interventional	NCT01828723

Fistula	6	Phase 1/Phase 2	Allogenic ASCs	Interventional	NCT01372969; NCT00999115
		Phase 1	Autologous ASCs	Interventional	NCT00992485
		Phase 2	Autologous ASCs	Interventional	NCT01011244
		Phase 3	Autologous ASCs	Interventional	NCT00475410
		Prospective	Autologous ASCs	Observational	NCT01020825

Liver cirrhosis	1		Autologous ASCs	Interventional	NCT01062750

Cardiovascular disease	4	Phase 1	Autologous SVFs	Interventional	NCT00442806; NCT00426868
		Phase 2	Autologous ASCs	Interventional	NCT01449032; NCT01216995

Peripheral vascular diseases and cardiovascular diseases	1	Phase 1/Phase 2	Autologous SVFs	Interventional	NCT01211028

Urinary incontinence	1	Phase 2	Autologous ASCs	Interventional	NCT01799694

Localized adverse reaction to administration of drug	1	Phase 1	Allogenic ASCs	Interventional	NCT01743222

Osteoarthritis	2	Phase 1	Autologous ASCs	Interventional	NCT01585857
		Phase 1/Phase 2	Autologous ASCs	Interventional	NCT01809769

Buerger's disease	1	Phase 1/Phase 2	Autologous ASCs	Interventional	NCT01302015

Brain injury	1	Phase 1/Phase 2	Autologous ASCs	Interventional	NCT01649700

Cerebellar ataxia	1	Phase 1/Phase 2	Allogenic ASCs	Interventional	NCT01649687

Spinal cord injury	2	Phase 1	Autologous ASCs	Interventional	NCT01274975; NCT01624779

Soft tissue mass removal	1		SVFs	Interventional	NCT01399307

Diabetic foot ulcer	1	Phase 1	Allogenic ASCs	Interventional	NCT02394886

Limb ischemia	2	Phase 1/Phase 2	Autologous ASCs	Interventional	NCT01257776; NCT01663376

Overweight	1	Prospective	Bone Grafts using ASCs and different scaffolds	Observational	NCT01218945

Rheumatoid arthritis	1	Phase 1/Phase 2	Allogenic ASCs	Interventional	NCT01663116

Degenerative arthritis	1	Phase 1/Phase 2	Autologous ASCs	Interventional	NCT01300598

Sepsis	1	Phase 1	Allogenic ASCs	Interventional	NCT02328612

Romberg's disease	1	Phase 2	Autologous ASCs	Interventional	NCT01309061

Depressed scar	1	Phase 2/Phase 3	Autologous ASCs	Interventional	NCT00992147

Healthy	1	Phase 1	Allogenic ASCs	Interventional	NCT01739530

ASCs, adipose-derived stem cells; SVFs, stromal vascular fraction.
